# The long-term management and outcomes of cloacal anomalies

**DOI:** 10.1007/s00467-014-2875-7

**Published:** 2014-09-13

**Authors:** M. Ashani Fernando, Sarah M. Creighton, Dan Wood

**Affiliations:** 1 Department of Urology, University College London Hospitals, 250 Euston Road, London, NW1 2PG UK; 2 Department of Women’s Health, University College London Hospitals, 250 Euston Road, London, NW1 2PG UK

**Keywords:** Cloacal anomalies, Long-term follow-up, Renal function, Vaginoplasty, Reconstruction

## Abstract

Cloacal anomalies occur when failure of the urogenital septum to separate the cloacal membrane results in the urethra, vagina, rectum and anus opening into a single common channel. The reported incidence is 1:50,000 live births. Short-term paediatric outcomes of surgery are well reported and survival into adulthood is now usual, but long-term outcome data are less comprehensive. Chronic renal failure is reported to occur in 50 % of patients with cloacal anomalies, and 26–72 % (dependant on the length of the common channel) of patients experience urinary incontinence in adult life. Defaecation is normal in 53 % of patients, with some managed by methods other than surgery, including medication, washouts, stoma and antegrade continent enema. Gynaecological anomalies are common and can necessitate reconstructive surgery at adolescence for menstrual obstruction. No data are currently available on sexual function and little on the quality of life. Pregnancy is extremely rare and highly risky. Patient care should be provided by a multidisciplinary team with experience in managing these and other related complex congenital malformations. However, there is an urgent need for a well-planned, collaborative multicentre prospective study on the urological, gastrointestinal and gynaecological aspects of this rare group of complex conditions.

## Introduction

**Fig. 1:**
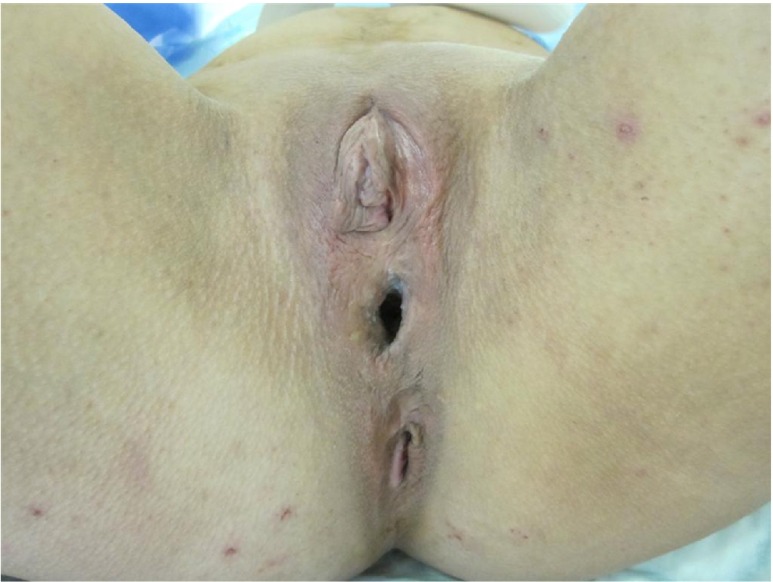
Photograph of an adult perineum in a female patient with a previously operated cloacal anomaly

Cloacal anomalies (persistent cloaca) are abnormalities of the urogenital sinus and anorectum; to date, their aetiology is still unknown (Fig. 1). These conditions are rare, although the true incidence is difficult to ascertain due to differences in classification and the inclusion of other rectal anomalies in some estimates. However, it is thought that the incidence of cloacal anomalies worldwide is approximately 1:50,000 live births [1].

Initial management focuses on anatomic reconstruction of the urinary and gastrointestinal system to achieve continence. Improved paediatric management strategies have increased patient survival into adult life. In order to provide appropriate advice, clinicians undertaking long-term management of adolescent and adult patients need to understand functional outcomes, including psychological and psychosexual health, as well as sexual function and reproductive potential.

At present, there is little- medium and long-term follow-up data available on this patient group. The aim of this review is to identify key uncertainties which arise in the management of adolescent and young adult women and to review the available medical literature in search of any supporting or informative evidence. Gaps in scientific knowledge were identified in order to suggest potential research opportunities.

## Methodology

The key clinical areas identified for the purposes of the review are:

1. Urinary continence
2. Renal function
3. Faecal continence
4. Sexual and reproductive function

A literature search was performed using the search terms “cloaca” and “anorectal”. In addition, citation lists from all identified articles were checked to ensure that all possible papers were included in the review.

The majority of papers included cloacal anomalies as part of a more mixed group of anorectal malformations. In some series, the data on cloacal anomalies were tabulated separately, but in others this was not done, making it impossible to identify outcomes in this subgroup. Finally, cloacal anomalies are usually divided into complex or high anomalies where the common channel is longer than 3 cm and low anomalies where the common channel is shorter than 3 cm. It is possible that poorer outcomes would occur in patients with a more complex anomaly; however, none of the authors of these papers classified their outcome data in this manner.

## Understanding cloacal anomalies

### Embryology

During fetal cloacal development the primitive hindgut (dorsal) and allantois (ventral) converge. The cloaca is an endodermal structure that is first apparent in the second week of gestation. At 4–6 weeks of gestation the urorectal septum descends, in the coronal plane, and separates the allantois from the hindgut, thereby creating the ventral urogenital membrane and the dorsal anal membrane, respectively. External genitalia have not developed at this stage. Failure of this process results in a common cloaca, i.e. with the urethra, vagina and the rectum all opening through a single, common channel [2]. The degree of failure of descent is variable, resulting in a wide spectrum of genital anomalies affecting the bladder and urethra, the vagina and the rectum and anus.

### Clinical classification

Multiple classification systems exist. The anatomical classification system introduced by Pena, outlined below, clarifies the term “cloacal anomaly” [3]. Therefore, the common term “persistent cloaca” implies a common channel as described above. The range of anomalies described is shown in Table 1.

**Table 1:** Classification of anorectal malformations

Female anorectal malformations
Perineal fistula
Vestibular fistula
Persistent cloaca
≤3 cm common channel
>3 cm common channel
Imperforate anus without fistula
Rectal atresia
Complex defects

Redrawn from Levitt and Pena [4], with permission

Associated anomalies are common and include:

1. Renal anomalies:
  common association with vesicoureteric reflux
  can occur in 33–83 % of cases, including dysplasia, fusion, ectopia, pelviureteric obstruction and duplication.

2. Gynaecological anomalies:
  Mullerian agenesis
  duplication, including bicornuate and didelphic uterus and vaginal septae
  sacral and spinal anomalies

3. Gastrointestinal anomalies:
  Trachea–oesophageal anomalies in 10 % of cases

The diagnosis may be made antenatally based on ultrasound imaging but can also be an unexpected finding at birth. Immediate management focuses on identification of the cloacal defect with appropriate resuscitation and stabilisation. Transfer to a specialist centre offering multidisciplinary care should follow immediately.

### Definitive reconstruction

The goals of reconstruction are to attain bladder, bowel, sexual and, ultimately, reproductive function. The length of the common channel (<3 cm and >3 cm) separates these patients into two groups.

Historically, patients with a diagnosis of cloacal anomaly underwent posterior sagittal anorecto–vagino–urethroplasty, with an associated high complication rate. In a study involving 54 patients, Levitt and Pena [4] identified rates of 25 % vaginal stenosis, 18 % urinary incontinence, six cases of urethrovaginal fistula, one ureteric injury and one total ischaemic necrosis of the vagina. In an attempt to reduce the complication rates and reduce the time spent dissecting the urethra from the vagina, total urogenital mobilisation (TUM) has been advocated.

TUM involves dissecting the vagina and urethra together away from the rectum, thus making the procedure technically easier and reducing the risk of vascular compromise. In a series of 11 patients who underwent TUM, Pena [5] noted no urethrovaginal fistula, acquired vaginal atresia or vaginal stricture at a follow-up of 1–14 months.

Longer common channels (>3 cm) pose a greater technical challenge with the requirement of a laparotomy as well as posterior sagittal incision; shorter channels may be treated through a perineal approach. In channels >5 cm it is impossible to mobilise the common channel to the perineum and, therefore, the channel is left as a urethra and the vagina is separated; ureteric damage is a risk. The vagina may be reconstructed utilising the Mullerian structures or, alternatively, an intestinal vagina can be formed. The long-term issues arising as a result of using the bowel in reconstructing the vagina make it the tissue of last resort.

### Pediatric outcomes

Based on a review of the medium-term outcomes of 22 girls operated on over a 5-year period at Great Ormond Street Hospital, Pena [6] reported a 23 % revision rate during the study period. Most of these patients had a long common channel. Urethral stenosis was noted in two of the patients with minor urogenital sinus revision required in one. Vaginal stenosis was noted in three patients, with no comment on further operative intervention, and five patients were reported to have anal stenosis. This is the largest single series published to date and clearly demonstrates the difficulty in finding published data on such patients.

### Long-term management considerations

Commonly, the patients are well supported in tertiary pediatric hospitals until they reach adolescence—thereafter, as with many urological and gynecological disorders, life-long care depends very much on identifying a centre with an interest in these adult patients. It is difficult to generalise a heterogenous population, and given the relative rarity of the condition, experience in management is confined to a small number of tertiary centres. Consequently, any ongoing evaluation of growth and physical and psychological health is difficult to undertake. In the following sections, we attempt to summarise and extrapolate the current literature based on urological, gynecological, obstetric and psychological indices.

### Urological anomalies

A structural anomaly of the renal tract is identified in a high proportion of this population, with reported rates as high as 83 % [7].

#### Upper tracts

A 20-year retrospective analysis completed in 2002 identified a number of important factors in renal function preservation and subsequent morbidity associated with renal failure [7]. The study cohort comprised 53 patients with cloaca and associated renal anomalies. Chronic renal failure was reported in 50 % of patients, with a glomerular filtration rate (GFR) of <80 mL/min per 1.73 m^2^; 17 % progressed to end-stage renal failure, with 6 % requiring transplantation and four patients dying of chronic renal failure. Of particular note is that 21 patients had a normal nadir creatinine during their first year of life. This is thought to be a positive prognostic indicator; however, in this population the nutritional status may have had an effect on the measurement of creatinine levels, leading to an underestimation. Patients with vesicoureteral reflux and a solitary kidney appeared to be at greatest risk. Consequently, regular ultrasound and functional imaging may be important in this group.

#### Lower urinary tract

Based on their experience in managing 490 cases of cloacal malformations, Levitt and Pena [8] suggest urinary continence occurs in 74 % of patients with channels <3 cm in length and in 28 % of patients with channels >3 cm —although the mean length of follow-up of this study group is not clear [9]. Matsui et al. [10] reviewed 11 children post-TUM with a median follow-up period of 72 months. Of these patients, six were continent, with three patients voiding spontaneously. All of these patients underwent post-operative urodynamic studies and all of them revealed either detrusor overactivity or a hypocontractile detrusor. Of the five patients with detrusor overactivity, two resolved spontaneously over the follow-up period. The observed continence rate was higher in patients with a stable detrusor. However, the cohort was too small to achieve statistical significance [8].

Urinary continence will depend on various factors, including bladder capacity, ability to catheterise, sphincter function and parental/social issues [10]. Long-term outcomes are not well-reported. In a retrospective review of 75 patients with anorectal anomalies, Gearhart and Jeffs [11] identified 15 patients with cloacal anomalies and noted that those patients with severe anomalies had worse continence scores. At a mean follow-up period of 26 years, 20 and 46 % % of the study population reported perfect/good continence and severe incontinence, respectively [11].

The early results of initial total urological reconstruction were suboptimal, with nearly 100 % incontinence rates reported [12]. In contrast, currently reported rates of spontaneous voiding after total urogenital mobilisation are reported to be 43–66 % [3, 8]. A proportion of the patients will be dry with the need to undertake clean intermittent catheterisation to empty. Difficulty in catheterisation, recurrent urinary tract infections or mechanical issues with catheters may lead to further reconstruction to improve bladder drainage [13].

A retrospective analysis of 141 (between 1959 and 1998) patients in a single surgeon practice [1] in Boston, Massachusetts identified spontaneous voiding in 83 (58 %) patients, intermittent catheterisation in 40 (28 %) patients and five (3 %) patients with urinary diversions. Five (3 %) patients were incontinent and thought to be candidates for further incontinence surgery. Only 24 patients in this study population were described as adults, and no information is given on the age range. This mixed group was accrued over a 39-year period, and it is unclear how much can be extrapolated from this population.

Braga et al. [14] studied 12 patients with a background of cloacal anomalies and reported that 66 % (8/12) had dry intervals of >4 h, with six of these undertaking clean intermittent catheterisation—five via a Mitrofanoff channel and one through the urethra—and two voiding spontaneously. The incontinence rate was quoted as 33 % (3/12), with one patient still awaiting reconstruction [14]. The varying surgical treatments reflect the complexity of lower tract reconstruction and the need to individualise treatment to attain the most successful functional outcome for the patient. There are as yet no definitive studies identifying the clinical progression of this patient population. Interval renal function should be formally assessed with annual serum biochemistry tests, and supplementary with chromium-EDTA GFR assays every 5 years have provided a useful monitor of change in our unit. Post-reconstruction, patients should have their upper tracts monitored to ensure anatomical and functional stability.

### Sexual and reproductive function

Gynaecological outcomes include menstruation, sexual function and fertility. The aim of reconstructive surgery should be to preserve a functional uterus—if present—and to provide a vagina allowing menstruation and later sexual activity. Unfortunately, there are limited outcome data on how well these objectives are achieved. Large studies of the surgical management of cloaca in infancy refer to a “significant” number of patients with gynaecological difficulties but do not provide details of management or long-term outcomes [3].

#### The uterus

There is a well-documented association between cloacal and Mullerian anomalies, with up to 30 % of girls having an associated Mullerian anomaly [3, 11]. These include Mullerian agenesis resulting in an absent uterus and/or vagina, as well as duplication anomalies including vaginal duplication and septae. It is very difficult to predict future uterine function before puberty as associated Mullerian anomalies are often not diagnosed until adolescence. The uterus is very small in childhood, and ultrasonography, magnetic resonance imaging and direct visualisation at laparoscopy or laparotomy have all been shown to be inaccurate [15].

At the onset of puberty, the uterus—if present—will grow and function. In a retrospective review of 41 post-pubertal patients with a cloaca, Warne et al. identified 68 % with a functioning uterus of whom 32 % were menstruating normally and 36 % presented with haematometra/haematocolpos [16]. Of note, six patients who had been identified as having an absent or vestigial uterus at birth yet presented with obstructed menstruation at puberty. Another study of 22 post-pubertal girls operated on for cloacal anomaly identified obstructed menstruation in nine girls (41 %) [17].

Neonatal reconstructive surgery for cloacal anomalies may not include vaginal reconstruction, and the vagina may re-stenose even if surgery is performed. Late presentation with obstructed menstruation at puberty due to absence of the vagina or stenosis can occur. Menstruation may be from a single obstructed uterus. However, the increased risk of duplication anomalies exists. Therefore, presentation of an adolescent with a painful pelvic mass should prompt investigation for a second obstructed horn.

It should be made clear to parents that the potential for uterine function cannot be assessed in childhood. These data suggest that where there is uncertainty, care should be taken when removing tissue with uterine potential. Regular monitoring, including imaging, during puberty will allow obstructed menstruation to be anticipated and managed. Cyclic abdominal pain with no menstruation should prompt an earlier ultrasound scan to identify obstructed menstruation. If more reconstruction is required, hormonal manipulation can control the menses until definitive repair can be undertaken [17].

#### The vagina

Vaginal reconstruction is usually undertaken in infancy at the time of initial reconstruction but can be deferred until later in childhood or adolescence. In some cases, the presence of hydrocolpos necessitates early vaginoplasty to prevent pelvic outlet obstruction. However, the timing of vaginoplasty in the absence of hydrocolpos is still debated, with no clear evidence base on either side. Supporters of early surgery cite the ease of a single procedure, as well as the benefits of a single anaesthetic and elimination of the risk of psychological trauma from surgery at puberty. Alternatively, early reconstruction carries the risk of late vaginal stenosis and obstructed menstruation has been described in 36–41 % of patients in this population [18, 19]. Late intervention is supported by some clinicians due to a potential decreased risk of complications compared to the prepubertal group. The authors of a recent retrospective analysis [20] concluded that the onset of puberty with the resultant oestrogenic effects on the vaginal tissues may improve healing, reducing the risk of re-stenosis as it would be a primary reconstructive procedure rather than a secondary one and that obstructed menstruation may act as a tissue expander. However, this cohort was heterogenous, with patients with cloacal anomalies comprising only 12 of 63 patients requiring vaginal reconstruction. In addition, the age at surgery or the length of follow-up of this cloacal subgroup of patients were not available.

A wide variety of vaginoplasty procedures have been employed in this patient group, including urogenital sinus mobilsation, insertion of skin flaps and intestinal substitution. Buccal mucosa has been introduced and could be a potential graft for selected patients needing vaginoplasty [21]. The surgical options depend on the anatomy and previous surgical history of the patient and surgeon preference. Post-operative dilation may reduce the risk of stenosis, although no data support this notion, and dilation should not be undertaken in a prepubertal girl. If there is no functional uterus, then vaginal reconstruction can be delayed until sexual activity is considered. The wide variety of presentation, management approach and surgical techniques makes long-term comparative outcome studies very difficult.

### Sexual function

To date, no studies have been published with the specific aim of assessing sexual function in women following surgery for cloacal anomalies. Available information is drawn from studies on larger cohorts of patients with a variety of diagnoses, including cloacal anomalies.

Sexual function in women is affected by both physical and psychological factors. Physical factors relevant in cloacal anomalies include vaginal stenosis, resulting in sex which is impossible or painful. In their retrospective review of 41 adolescents with cloacal anomalies, Warne et al. [16] found that 18 patients (44 %) had an adequate vagina for intercourse and six (15 %) required revision vaginal surgery to facilitate intercourse. However, the remainder of the group (54 %) had not been evaluated, and it is possible that more patients would have stenosis. This study did not examine sexual function. Perineal scarring from genital reconstructive surgery has been found to reduce sexual sensitivity and sexual satisfaction in other congenital genital anomalies [16]. It is likely that the same will apply in the case of reconstructive surgery for the cloaca, but this has never been studied. Psychological factors affecting sexual function include poor body esteem and the impact of chronic illness. While these factors have been studied as a cause of psychological morbidity in children with anorectal malformations, including those of the cloaca, there is little relevant literature on adult patients. A study of adults with anorectal malformations (of whom one third of the female patients had a cloaca) found reduced body esteem when compared to the normal population [11]. This study also found that urinary incontinence was associated with raised sexual anxiety and fear of sexual relationships. As the most severe incontinence was found in the subgroup with cloacal anomalies, it is likely that these patients also suffered from sexual difficulty.

### Fertility

Several factors can impact the ability of a woman with a cloacal anomaly to bear children. These include:

- The presence of Mullerian anomalies. If the uterus is absent, then pregnancy is not possible. If the uterus is unicornuate, bicornuate or didelphic, then the risks of miscarriage and preterm birth are significantly increased, although the exact magnitude is uncertain. Extreme prematurity carries high risks of physical and neurological damage to the infant.
- Multiple previous surgical procedures leading to mechanical damage to the Fallopian tubes
- Vaginal stenosis resulting in the inability to have intercourse or intercourse that is painful
- Factors impacting upon general health, such as renal failure and other associated anomalies. Renal impairment may be due to apparent ureteric obstruction by the gravid uterus on a background of reduced functional reserve.

There are very few reports of successful pregnancies in women with cloacal anomalies. The largest study of 339 patients with unspecified follow-up reported only 2 pregnancies both delivered vaginally but neither was in the “complex” cloaca group. Only 5 further cases were identified by this review over a period of 20 Years [22–26]. Reported complications have included miscarriage, preterm labour and worsening of renal function. A case report in 2008 identified a patient with a cloacal anomaly who underwent an emergency caesarean section at 27 weeks and 4 days with a background of 5 previous miscarriages [22]. In a retrospective analysis of 24 patients of whom 17 are sexually active and 6 are parous, 1 patient had a vaginal delivery [1].

It is difficult therefore to offer any guidance to women based on such [1] a small number of cases. It is likely that there have been other successful pregnancies so far unreported in the literature. There is no data on the number of women who have tried unsuccessfully to conceive, those who have had fertility input and those who have lost a pregnancy from miscarriage or early preterm birth.

Increasing numbers of young women with cloacal anomalies survive into adulthood and have the same reproductive aspirations as their peers. This will mean more women in this group seeking both fertility treatment and pregnancy care. Whilst it is difficult to draw specific guidelines given the lack of data, pregnancy care could sensibly be based on the guideline for the multidisciplinary management of pregnancy in women with bladder extrophy [27].

### Gastrointestinal anomaly

The mainstay of management stems from the principles of maintaining appropriate bowel length to ensure short gut syndrome is avoided and growth is possible [28]. There is some adaptation of the intestinal tract as the child grows.

Small lengths of colon that were incorporated into a pull-through have been noted to grow as stool passes through it. Pena [29] followed 25 patients with anterior and posterior sagittal repairs for 23 years. At end of the follow-up, three patients (12 %) were completely continent and four (16 %) patients still soiled [29]. Eleven patients (44 %) were continent with a bowel management programme, and four (16 %) patients were incontinent and were candidates for a bowel management programme. Two patients (8 %) were converted to an ileostomy, and one patient (4 %) refused a bowel management programme. These outcomes are similar to the Boston cohort in which 82/141 (52 %) patients had spontaneous bowel movements and satisfactory continence. Levitt et al. [8] suggested that channel length may influence outcomes. In their study, 38/141 patients (26 %) remained on a bowel management programme; faecal continence was achieved in 66 % of these with a channel length of <3 cm, which fell to 36 % in those with a channel length of >3 cm. Among the remaining patients, seven (4 %) continued to soil; however, these are described as children, and the follow-up period is unclear.

## Conclusion

This review has identified the lack of available long-term follow-up data for women with cloacal anomalies. Pediatric care usually takes place in specialised centres with surgical and medical expertise. However—as for many complex chronic conditions—adult care is poorly organised. Patients are referred for adult follow-up to general colorectal or urology clinics at local hospitals, and gynaecological care is not usually offered. This leads to fragmented medical care of variable quality and also to a lack of ability to collect information on long-term outcomes. It is essential that adolescents with cloacal anomalies transition to specialist teams with appropriate expertise. Long-term adult follow-up should also remain under the care of specialised multidisciplinary teams familiar with the management of these and other related complex congenital malformations, and clinical decisions should be individualised. However, there is an urgent need for a well-planned, collaborative multi-centre prospective study to define outcomes and plan follow-up strategies to optimise health.

## Multiple choice questions: (answers are provided following the reference list)

1. In embryology:
  - the cloaca is separated into the urogenital and anorectum between 4 and 6 weeks of gestation;
  - the allantois is not related to the primitive cloaca;
  - the ureters drain separately from the primitive hindgut;
  - in the female the urethra is derived from both the urogenital groove andthe urogenital canal.

2) Cloacal anomalies are not associated with the following:
  - duplication, including bicornuate and didephic uterus
  - fibular agenesis;
  - sacral and spinal anomalies;
  - trachea–oesophageal anomalies.

3) Which following statements are true:
  - vaginal delivery is not possible with a cloacal anomaly;
  - a shorter common channel has been shown to lead to less sexual dysfunction;
  - continence rates for both urine and stool fall by 50 % if the channel length is >3 cm;
  - following good surgery in infancy, revision rates are <2.
